# Multisite rTMS combined with cognitive training modulates effective connectivity in patients with Alzheimer's disease

**DOI:** 10.3389/fncir.2023.1202671

**Published:** 2023-09-05

**Authors:** Yuanyuan Qin, Li Ba, Fengxia Zhang, Si Jian, Tian Tian, Min Zhang, Wenzhen Zhu

**Affiliations:** ^1^Department of Radiology, Tongji Hospital, Tongji Medical College, Huazhong University of Science and Technology, Wuhan, Hubei, China; ^2^Department of Neurology, Tongji Hospital, Tongji Medical College, Huazhong University of Science and Technology, Wuhan, Hubei, China; ^3^Department of Rehabilitation, RenMin Hospital of Wuhan University, Wuhan, Hubei, China

**Keywords:** repetitive transcranial magnetic stimulation, cognitive training, Alzheimer's disease, resting-state fMRI, effective connectivity

## Abstract

**Purpose:**

To investigate the effective connectivity (EC) changes after multisite repetitive transcranial magnetic stimulation (rTMS) combined with cognitive training (COG).

**Method:**

We selected 51 patients with mild or moderate Alzheimer's disease (AD) and delivered 10 Hz rTMS over the left dorsal lateral prefrontal cortex (DLPFC) and the lateral temporal lobe (LTL) combined with COG or sham stimulation for 4 weeks. The selected AD patients were divided into real (real rTMS+COG, *n* = 11) or sham (sham rTMS+COG, *n* = 8) groups to undergo neuropsychological assessment, resting-state fMRI, and 3D brain structural imaging before (T0), immediately at the end of treatment (T4), and 4 weeks after treatment (T8). A 2 × 3 factorial design with “time” as the within-subjects factor (three levels: T0, T4, and T8) and “group” as the between-subjects factor (two levels: real and sham) was used to investigate the EC changes related to the stimulation targets in the rest of the brain, as well as the causal interactions among seven resting-state networks based on Granger causality analysis (GCA).

**Results:**

At the voxel level, the EC changes from the left DLPFC out to the left inferior parietal lobe and the left superior frontal gyrus, as well as from the left LTL out to the left orbital frontal cortex, had a significant group × time interaction effect. At the network level, a significant interaction effect was identified in the increase in EC from the limbic network out to the default mode network. The decrease in EC at the voxel level and the increase in EC at the network level were both associated with the improved ability to perform activities of daily living and cognitive function.

**Conclusion:**

Multisite rTMS combined with cognitive training can modulate effective connectivity in patients with AD, resulting in improved ability to perform activities of daily living and cognitive function.

## Highlights

- Multisite rTMS combined with cognitive training has been shown to be potentially effective in patients with early-stage Alzheimer's disease.
- The effective connectivity changes associated with this new non-drug adjuvant intervention were investigated.
- The decrease in effective connectivity at the voxel level and the increase at the network level were both associated with improved ability to perform activities of daily living and cognitive function.

## Introduction

Alzheimer's disease (AD) is an irreversible neurodegenerative disorder with recent understanding as a disconnection syndrome. The functional connectivity of large-scale networks is progressively disrupted during disease progression (Gomez-Ramirez and Wu, 2014). As pharmacotherapy for AD is currently limited, attention has been paid to non-drug adjuvant interventions such as repetitive transcranial magnetic stimulation (rTMS). Using a pulsed magnetic field to create a current in the human brain, rTMS can produce a persistent effect on the cortical synapse function after stimulation (Huang et al., 2017). The frequency of pulse applications during rTMS and the stimulation target are two prominent parameters for evaluating the after-effects of rTMS. Traditionally, a high frequency induces an excitation effect while a low frequency induces the opposite (Riedel et al., 2019). High-frequency rTMS delivered at 10 Hz significantly improved the cognitive function in AD patients but low-frequency rTMS delivered at 1 Hz did not (Ahmed et al., 2012). For the stimulation target, single-site rTMS on the left dorsal lateral prefrontal cortex (DLPFC) was shown to be potentially effective in patients with AD (Level C of evidence) (Di Lazzaro et al., 2021).

Recently, multisite rTMS combined with cognitive training, also called “rTMS-COG therapy,” has been shown to be potentially effective in AD patients at the early stage (Level B of evidence) (Di Lazzaro et al., 2021). Using the NeuroAD system, 6-site (left and right DLPFCs, left and right parietal cortices, Broca's area, and Wernicke's area) rTMS combined with cognitive training improved apathy and cognitive functions, including memory and language, in AD (Lee et al., 2016; Suarez Moreno et al., 2022). However, upon directly comparing a single site (only left DLPFC) and multisite (the same as the aforementioned 6-site approach) rTMS procedure, both approaches performed similarly within a short treatment period (Alcala-Lozano et al., 2018). The discrepancy is presumably due to the use of rTMS alone. Another double-site (right middle frontal gyrus and right inferior parietal lobule) rTMS procedure that did not combine cognitive training failed to validate the multi-target focused rTMS hypothesis in young healthy participants (Feng et al., 2021). A possible explanation would be that coupling multisite stimulation with cognitive training enhances the treatment effect (Nguyen et al., 2018). The combination of multi-target stimulation and cognitive training has shown to be a promising clinical application prospect. However, the neurobiological effects of rTMS-COG therapy need to be further expounded.

Resting-state functional magnetic resonance imaging (rs-fMRI) is an appropriate tool for evaluating the neurobiological effects of neurostimulation. rTMS has been approved for its ability to modulate local activity in a remote area that is functionally connected to cortical stimulation targets (Aceves-Serrano et al., 2022; Qin et al., 2022). However, the causal interactions between the stimulation targets and other brain regions have yet to be explored. To our knowledge, functional connectivity (FC) is defined as the temporal correlation between two remote areas that are computationally efficient but undirected; effective connectivity (EC) further provides direction information about these associations (Deshpande and Hu, 2012). Granger causality analysis (GCA) determines whether the activity in brain region X engages in directed interaction with the activity in region Y, or vice versa (de Graaf et al., 2009). GCA on rs-fMRI data enabled us to investigate EC based on multiple linear regression (Deshpande and Hu, 2012) and determine the positive or negative influences of the stimulation targets on the rest of the brain after rTMS stimulation.

Besides the commonly known dysfunction of the default mode network (DMN) (Greicius et al., 2004), AD is also affected by other large-scale functional brain networks, such as the executive control network (ECN) and frontoparietal network (FPN) (Liu et al., 2012; Zhao et al., 2018). The DLPFC, associated with working memory and attention, is an important node of the ECN and the FPN, and the lateral temporal lobe (LTL) nearest to the hippocampal formation (HF) is an important node of the DMN. In this study, we chose the left DLPFC and left LTL as double-site stimulation targets, combined with six types of cognitive training tasks (Zhang et al., 2019; Qin et al., 2022). The study aimed to investigate the EC changes associated with stimulation targets on the rest of the brain, as well as the causal interactions among resting-state networks (RSNs) after multisite rTMS-COG therapy.

## Materials and methods

The study was approved by the Ethics Committee of Tongji Hospital (Wuhan, China; Chinese Clinical Trail Registry Registration number: ChiCTR-INR-16009227). Written informed consent was obtained from all participants before enrollment according to the Declaration of Helsinki.

### Participants, study design, and intervention

Patients with AD were recruited in this study based on the National Institute of Neurological Disorders and Stroke-Alzheimer Disease and Related Disorders (NINCDS-ADRDA) criteria (Dubois et al., 2007). None of the participants had any history of head injury, stroke, depression, or tumor. All the patients had taken medicine such as acetylcholinesterase inhibitors (Donepezil) or N-Methyl-D-aspartate receptor antagonists (memantine) for at least 3 months at a stable dosage. In total, 51 participants with mild or moderate AD (clinical dementia rating ≤ 2) were recruited and randomly assigned into a real rTMS with cognitive training (*n* = 26) group or a sham group (only receiving cognitive training, *n* = 25) using a web-based randomization generator (http://www.jerrydallal.com/random/randomize.htm).

Before treatment, all patients underwent neuropsychological assessment and MRI scanning (T0). Then, the real rTMS or sham stimulation was repeated five times from Monday to Friday for 4 weeks. Another MRI scan and neuropsychological assessment were performed on Saturday morning at the end of the 4-week treatment (T4). To evaluate the long-time effect, an MRI scan and neuropsychological assessment were followed up 4 weeks after the end of treatment (T8). All neuropsychological measures before and after the treatment were assessed by a specialist with over 20 years of experience who was blinded to the allotment.

In the real group, rTMS was conducted in combination with cognitive training for up to 1 h each day. A butterfly coil (MCF-B65) with an inner diameter of 35 mm was used for the rTMS treatment, and the treatment was guided by an optical navigation system (Magventure, Germany). High-frequency rTMS pulses were delivered separately to the stimulation target first in the left DLPFC (Talairach coordinates: X = −35, Y = 24, Z = 48) and then in the left LTL (Talairach coordinates: X = −60, Y = −15, Z = −15). The following parameters were used: 20 trains (5 s duration at 10 Hz with an inter-train interval of 25 s), 100% resting motor threshold (RMT), 1,000 pulses, and 10 min in total for each target, and there were no maintenance sessions. The protocol in the sham condition was the same as that in the real condition, except that the coil was positioned with the lateral edge of one wing touching the scalp at 90°(Pascual-Leone et al., 1996). All participants were asked if they had any symptoms of discomfort after the stimulation.

The cognitive training was completed on an iPad tablet (version 9.1; Apple, USA) with several cognitive tasks selected by an experienced cognitive therapist. The memory tasks were completed during rTMS stimulation, while the other tasks, including attention tasks, mathematical calculations, agility drills, language tasks, and logic thinking tasks, were practiced after the stimulation ended (Zhang et al., 2019). Please refer to our previous article for detailed information.

### MRI data acquisition

The MRI data were acquired using a 3T scanner (Discovery 750, GE Healthcare) using a 32-channel head coil. To minimize head motion and reduce scanner noise, tight but comfortable foam padding and earplugs were used. During the resting-state fMRI scan, participants were instructed to close their eyes, not fall asleep, and not think of anything in particular. Gradient-echo echo-planar imaging (EPI) sequence was used to acquire fMRI images with the following parameters: repetition time/echo time (TR/TE) = 2000/30 ms, flip angle = 90°, matrix = 64 × 64, field of view = 240, slice thickness = 4 mm, interleaved acquisition, 29 axial slices in total, and 240 timepoints. A sagittal T1-weighted structure image was also acquired using a 3D brain-volume (3D-BRAVO) sequence with the following parameters: TR/TE = 8.2/3.2 ms, TI = 450 ms, slice gap = 1 mm, matrix = 256 × 256, flip angle = 8°, and voxel size = 1 × 1 mm.

### MR data preprocessing

The resting-state fMRI data were preprocessed using SPM8 (http://www.fil.ion.ucl.ac.uk/spm/). The first 10 timepoints of the rs-fMRI data were discarded, leaving the remaining 230 timepoints for slice timing and motion correction (threshold: translational or rotational motion parameters lower than 2 mm or 2°). The normalization included the following steps: (1) structural images were linearly coregistered to the mean functional image; (2) the transformed structural images were segmented, and then the gray matter was non-linearly coregistered to the Montreal Neurological Institute (MNI) space; and (3) the motion-corrected functional volumes were normalized to the MNI space using the parameters estimated during the non-linear coregistration. The functional images were resampled into a voxel size of 3 × 3 × 3 mm^3^ and then smoothed using a Gaussian kernel of 8 × 8 × 8 mm^3^ full width at half-maximum (FWHM). Finally, functional images were band-pass-filtered with a frequency from 0.01 to 0.1 Hz, and several nuisance covariates (24 head motion parameters, averaged signal from white matter, cerebrospinal fluid, and global signal) were regressed out by performing multiple linear regression analysis.

A 6 mm radius sphere centered on stimulation targets, that is, the left DLPFC and the left LTL, was used as the seed region. The coordinates were the same as those used for the rTMS treatment. Bivariate first-order coefficient-based voxel-wise GCA was performed to explore the influence of the stimulation target on the rest of the brain using the REST toolbox (http://www.restfmri.net).

Resting-state fMRI networks were identified by using the Group ICA program of the fMRI toolbox (http://www.nitrc.org/projects/cogicat/). Seven classic networks, including a visual network, a sensorimotor network, a dorsal attention network, a salience/ventral attention network, a limbic network, a control network, and a default mode network (DMN), were used as the networks of interest, which were obtained by a clustering approach across the cerebral cortex using resting-state functional connectivity MRI from 1,000 healthy subjects (Yeo et al., 2011). The generated network components were exported to calculate the inter-network effective connectivity.

### Statistics

The normal distribution of the data was determined by performing the Kolmogorov–Smirnov test. The χ^2^ test or Fisher's exact test was performed to compare the categorical variables. An independent t-test or the Mann–Whitney test was performed for the quantitative parameters. EC changes at the voxel and network level were analyzed using a flexible factorial design installed in SPM12. We considered a 2 × 3 factorial design with “time” as the within-subjects factor (three levels: T0, T4, and T8) and “group” as the between-subjects factor (two levels: real rTMS + COG and sham rTMS + COG). If the group × time interaction was significant, *post-hoc* analysis was performed to explore the simple effects of time and group with the least significant difference (LSD) correction. All behavioral measures were analyzed using the IBM Statistical Package for the Social Sciences (SPSS) version 25, and graphs were made in GraphPad Prism 8. The relationship between EC changes and behavioral changes before and after the intervention was explored by performing a partial correlation analysis. Sex, age, education level, CDR, and disease duration were set as covariates in all tests.

## Results

**Figure 1:**
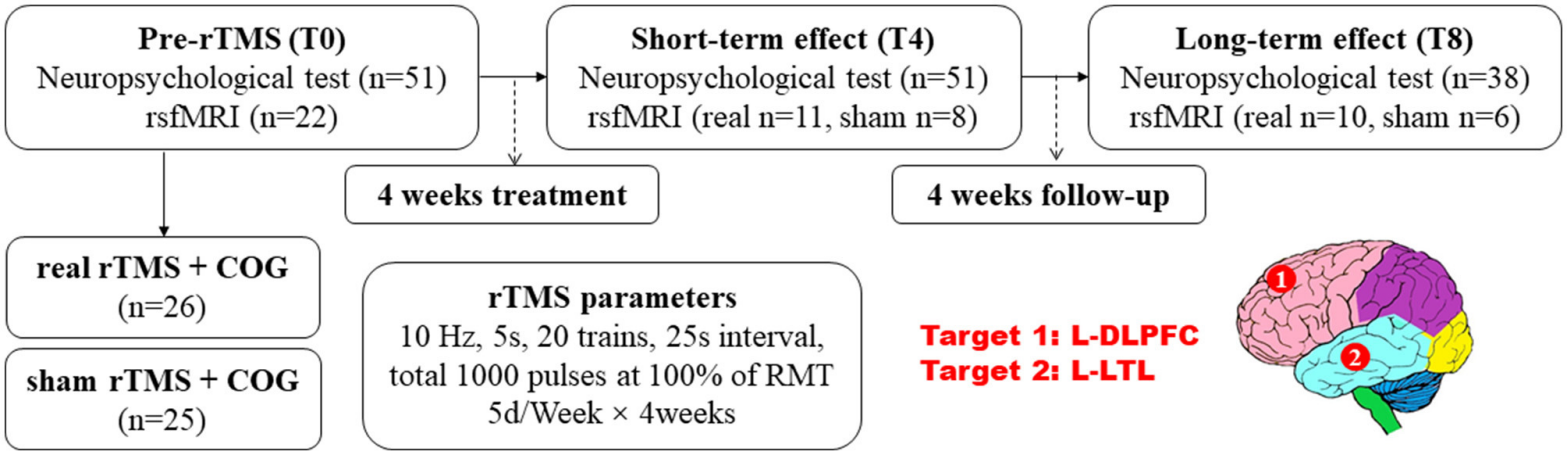
Experiment design: the evaluation of neuropsychological test scores and resting-state functional MRI before and after rTMS application, as well as the rTMS parameters and targets. rTMS, repetitive transcranial magnetic stimulation; COG, cognitive training; DLPFC, dorsal lateral prefrontal cortex, and LTL, lateral temporal lobe; L, left.

All the 51 patients enrolled received pre- and post-neuropsychological tests, with 26 allocated to the real rTMS + COG group and 25 to the sham rTMS + COG group, in which only 22 of them underwent pre-treatment MRI scan because the family members of the others refused. At the end of 4 weeks' treatment (T4), three patients in real group missed the MRI scan, with 11 patients in real group and 8 patients in sham group. At timepoint T8, one patient in real group refused to be scanned again. Two patients in sham group missed the scan time because of damage to the MRI instrument on the day of the scheduled. Figure 1 shows the flow diagram of this study and the detailed rTMS parameters.

Before treatment, there were no significant differences in sex, age, education level, course of the disease, and clinical dementia rating (CDR) between the real and sham groups (*p* > 0.05). Table 1 shows the detailed demographic characteristics. As for the alteration of neuropsychological scores, the MMSE score increment at T4 in the real group significantly differed from that of the sham group (*p* = 0.04). The activities of daily living (ADL) score decrease in the real group significantly differed from that in the sham group at T4 (*p* = 0.01) and T8 (*p* = 0.02). As for the Montreal Cognitive Assessment (MoCA), Alzheimer's Disease Assessment Scale-Cognitive Subscale (ADAS-cog), and the auditory verbal learning test (AVLT) changes, there were no significant differences between the real and sham groups at either T4 or T8 (*p* > 0.05).

**Table 1:** Demographic characteristics of the real and sham groups.

	**real rTMS + COG**	**sham rTMS + COG**	**t/Z/χ^2^**	***P***
Sex (male/female, *n*)	2/9	3/5	0.26	0.61
Age (yrs.)	67.36 ± 6.98	66.25 ± 8.07	0.32	0.75
Education (yrs.)	12.27 ± 1.79	11.50 ± 2.83	0.73	0.48
Course of disease (yrs.)	3.64 ± 1.96	3.63 ± 1.51	0.01	0.99
CDR (0.5/1/2)	3/4/4	0/5/3	2.85	0.24
MMSE T4-T0	3.00 ± 1.55	1.38 ± 1.51	−2.06	**0.04**
T8-T0	2.40 ± 1.58	1.50 ± 3.02	−1.16	0.25
MoCA T4-T0	2.64 ± 2.58	2.13 ± 2.36	−0.17	0.87
T8-T0	2.40 ± 2.27	2.00 ± 3.85	−0.55	0.59
ADAS-cog T4-T0	−3.79 ± 3.50	−0.96 ± 2.59	−1.78	0.08
T8-T0	−4.13 ± 2.26	−1.80 ± 4.14	−1.12	0.26
AVLT T4-T0	4.13 ± 3.80	1.45 ± 4.63	−0.71	0.48
T8-T0	8.75 ± 11.30	−2.86 ± 8.57	−1.70	0.09
ADL T4-T0	−2.73 ± 1.62	−0.88 ± 0.83	−2.59	**0.01**
T8-T0	−2.89 ± 2.20	1.00 ± 2.76	−2.26	**0.02**

rTMS, repetitive transcranial magnetic stimulation; COG, cognitive training; CDR, Clinical Dementia Rating; MMSE, Mini-Mental State Examination; MoCA, Montreal Cognitive Assessment; ADAS-cog, Alzheimer's Disease Assessment Scale-Cognitive Subscale; AVLT, auditory verbal learning test–HuaShan version; ADL, activities of daily living.

### EC changes at the voxel level

**Figure 2:**
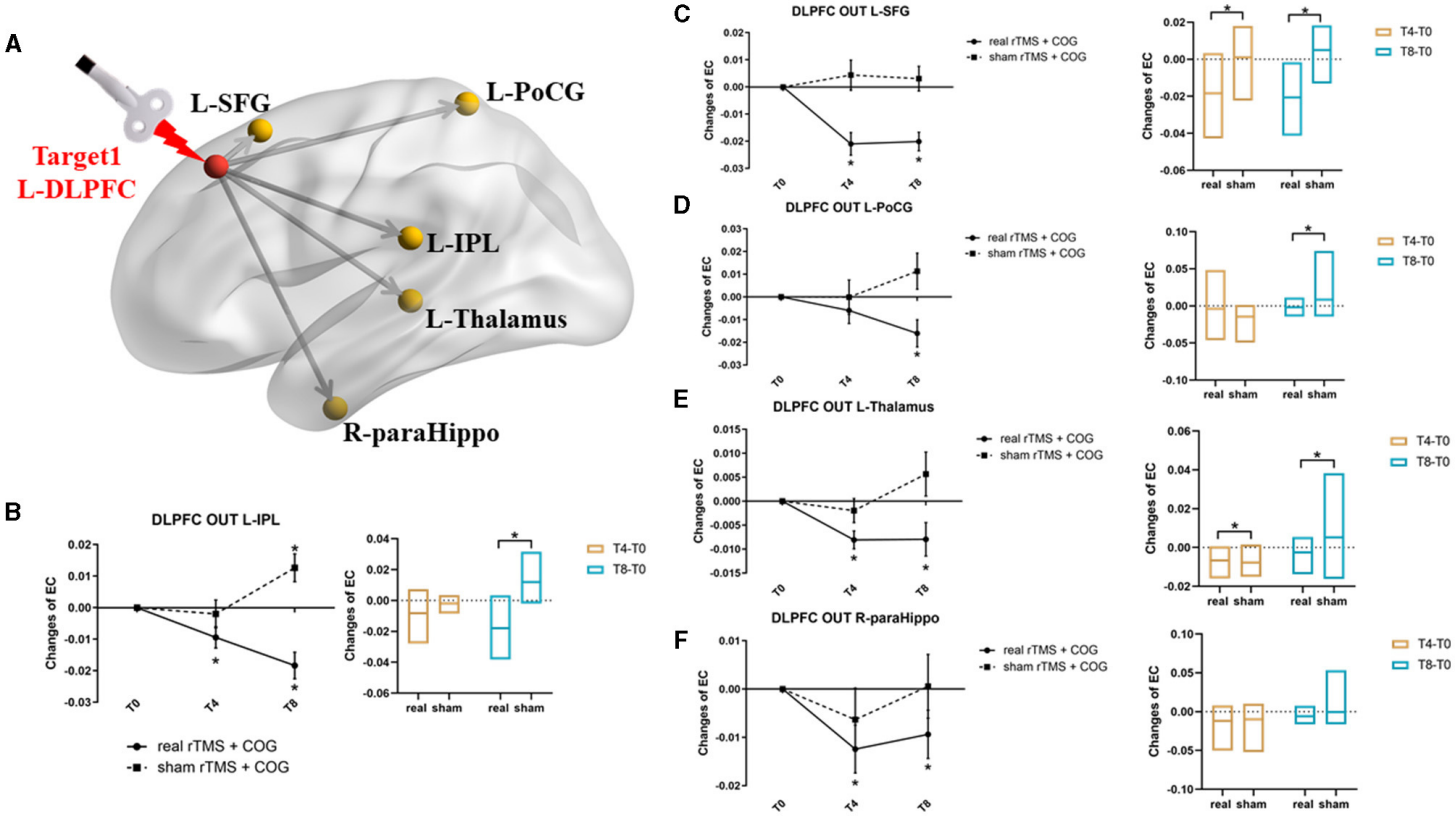
Results of EC changes from the left DLPFC out to the rest of whole brain at voxel level. **(A)** The pattern diagram showing the stimulation site (left DLPFC) and the five brain regions with significant EC changes. **(B–F)** Line and bar charts showing the *post-hoc* analysis results from left DLPFC out to left IPL **(B)**, left SFG **(C)**, left PoCG **(D)**, left thalamus **(E)**, and right parahippocampus **(F)**. *Indicates *p* < 0.05, FWE corrected. L, left; R, right; DLPFC, dorsal lateral prefrontal cortex; IPL, inferior parietal lobe; SFG, superior frontal gyrus; PoCG, postcentral gyrus; paraHippo, parahippocampus, EC, effective connectivity.

In general, the left DLPFC seed from the GCA analysis showed significant causal outflow to five clusters (*p* < 0.05, FWE corrected), that is, the left inferior parietal lobe (IPL), the left superior frontal gyrus (SFG), the left postcentral gyrus (PoCG), the left thalamus, and the right parahippocampus (Figure 2A). However, the reverse influence after rTMS-COG treatment was not significant (*p* > 0.05).

Specifically, a significant group × time interaction effect (F = 14.057, *p* = 0.000) was observed for EC changes from the left DLPFC to the left IPL. *Post-hoc* analysis indicated that, compared with the baseline, high-frequency rTMS + COG produced a significant reduction in EC at timepoints T4 (*p* = 0.010) and T8 (*p* = 0.001), while sham rTMS + COG produced a significant increase in EC at timepoint T8 (*p* = 0.024) (Figure 2B, line chart). The EC changes differed significantly between the real and sham groups at T8 (*p* = 0.001) but not at T4 (*p* = 0.115) (Figure 2B, bar chart).

In terms of EC changes from the left DLPFC to the left SFG, a significant time × group effect was also identified (F = 10.766, *p* = 0.001). Compared with the baseline, high-frequency rTMS + COG produced a significant reduction in EC at timepoints T4 (*p* = 0.000) and T8 (*p* = 0.000) (Figure 2C, line chart). The EC changes differed significantly between the real and sham groups at both T4 (*p* = 0.003) and T8 (*p* = 0.002) (Figure 2C, bar chart).

For EC changes from the left DLPFC to the left PoCG, only the time main effect was significant (F = 4.053, *p* = 0.035). High-frequency rTMS + COG produced a significant reduction in EC at timepoint T8 (*p* = 0.013) but not at timepoint T4 (*p* = 0.164) (Figure 2D, line chart). The EC changes differed significantly between the real and sham groups at T8 (*p* = 0. 013) but not at T4 (*p* = 0.285) (Figure 2D, bar chart).

The EC changes from the left DLPFC to the left thalamus also showed a significant time main effect (F = 3.579, *p* = 0.049). The real rTMS + COG produced a significant reduction in EC at timepoints T4 (*p* = 0.001) and T8 (*p* = 0.024) (Figure 2E, line chart). The EC changes differed significantly between the real and sham groups at both T4 (*p* = 0.046) and T8 (*p* = 0.025) (Figure 2E, bar chart).

The EC changes from the left DLPFC to the right parahippocampus also showed a time main effect (F = 5.704, *p* = 0.012). Compared with the baseline, the high-frequency rTMS + COG produced a significant reduction in EC at timepoints T4 (*p* = 0.017) and T8 (*p* = 0.047), while the results of the sham group were not significant at either T4 or T8 (Figure 2F, line chart). The EC changes between the real and sham groups also showed no difference at either T4 (*p* = 0.247) or T8 (*p* = 0.140) (Figure 2F, bar chart).

**Figure 3:**
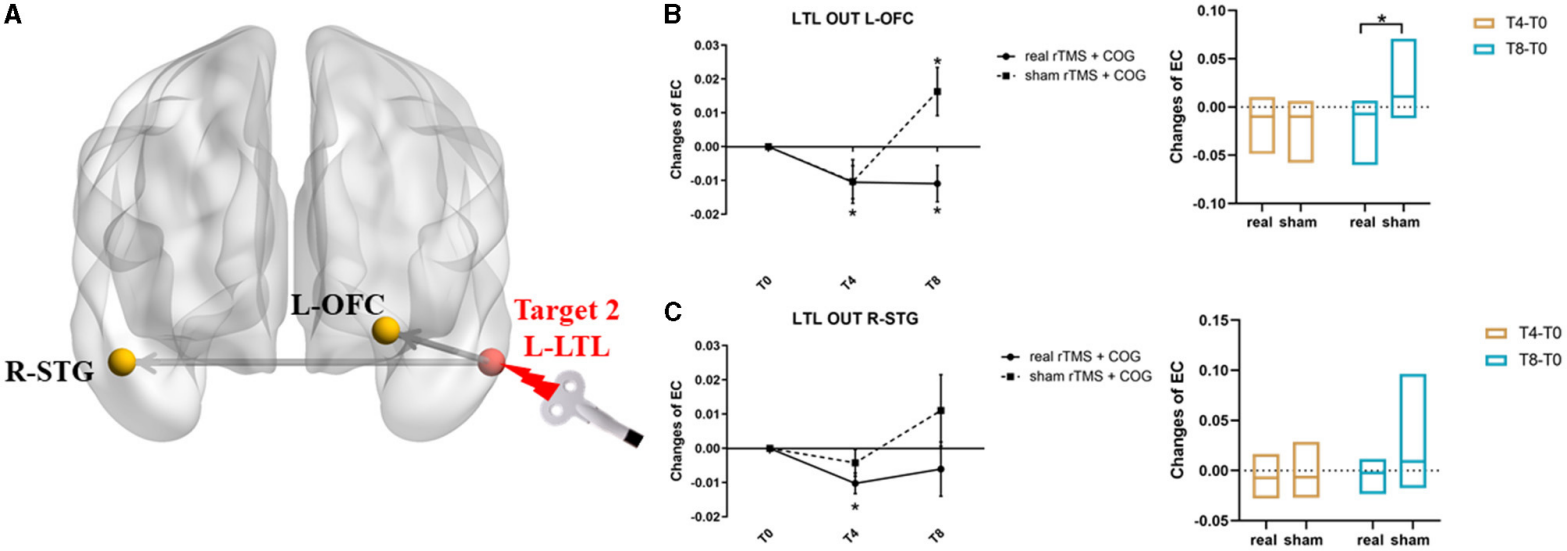
Results of EC changes from the left LTL out to the rest of whole brain at voxel level. **(A)** The pattern diagram showing the stimulation site (left LTL) and the two brain regions with significant EC changes. **(B, C)** Line and bar charts showing the *post-hoc* analysis results from left LTL out to left IOFC **(B)** and right STG **(C)**. *Indicates *p* < 0.05, FWE corrected. L, left; R, right; LTL, lateral temporal lobe; OFC, orbital frontal cortex; STG, superior temporal gyrus; EC, effective connectivity.

The left LTL seed from the GCA analysis revealed two clusters with significant causal outflow (*p* < 0.05, FWE corrected), that is, the left orbital frontal cortex (OFC) and the right superior temporal gyrus (STG) (Figure 3A), with no significant reverse influence (*p* > 0.05). The EC changes from the left LTL out to the left OFC showed significant time × group interaction (F = 4.198, *p* = 0.032). Compared with the baseline, the high-frequency rTMS + COG produced a significant reduction in EC at timepoints T4 (*p* = 0.030) and T8 (*p* = 0.037). The sham group showed no significant difference at T4 (*p* = 0.072), but it produced a significant increase in EC at T8 (*p* = 0.024) (Figure 3B, line chart). The EC changes between the real and sham groups differed significantly at T8 (*p* = 0.008) but not at T4 (*p* = 0.491) (Figure 3B, bar chart).

In terms of the EC changes from the left LTL to the right STG, the time main effect was significant (F = 7.442, *p* = 0.016). The real rTMS + COG produced a significant reduction in EC at timepoint T4 (*p* = 0.004), but it showed no significant changes at T8 (*p* = 0.232). The sham group showed no significant changes at either T4 (*p* = 0.155) or T8 (*p* = 0.159) (Figure 3C, line chart). The EC changes between the real and sham groups showed no significant difference at either T4 (*p* = 0.138) or T8 (*p* = 0.121) (Figure 3C, line chart). Table 2 summarizes the voxel-level results of EC changes for directional influence to and from the left DLPFC and left LTL seeds.

**Table 2:** 2 × 3 flexible factorial design results of effective connectivity changes.

	**Cluster size**	**Peak MNI coordinate**		**Peak intensity**	
DLPFC → L-IPL (group × time)	10	−60	−30	21	22.4392
DLPFC → L-SFG (group × time)	12	−24	18	57	25.8903
DLPFC → L-PoCG (time main effect)	5	−21	−48	6	21.094
DLPFC → L-Thalamus (time main effect)	6	−9	−30	0	27.6886
DLPFC → R-parahippocampus (time main effect)	14	24	−6	−36	30.9024
LTL → L-OFC (group × time)	40	−27	60	−12	33.3441
LTL → R-STG (time main effect)	38	48	12	−21	58.5413

L, left; R, right; DLPFC, dorsal lateral prefrontal cortex; IPL, inferior parietal lobe; SFG, superior frontal gyrus; PoCG, postcentral gyrus; LTL, lateral temporal lobe; OFC, orbital frontal cortex; STG, superior temporal gyrus.

### EC changes at the network level

**Figure 4:**
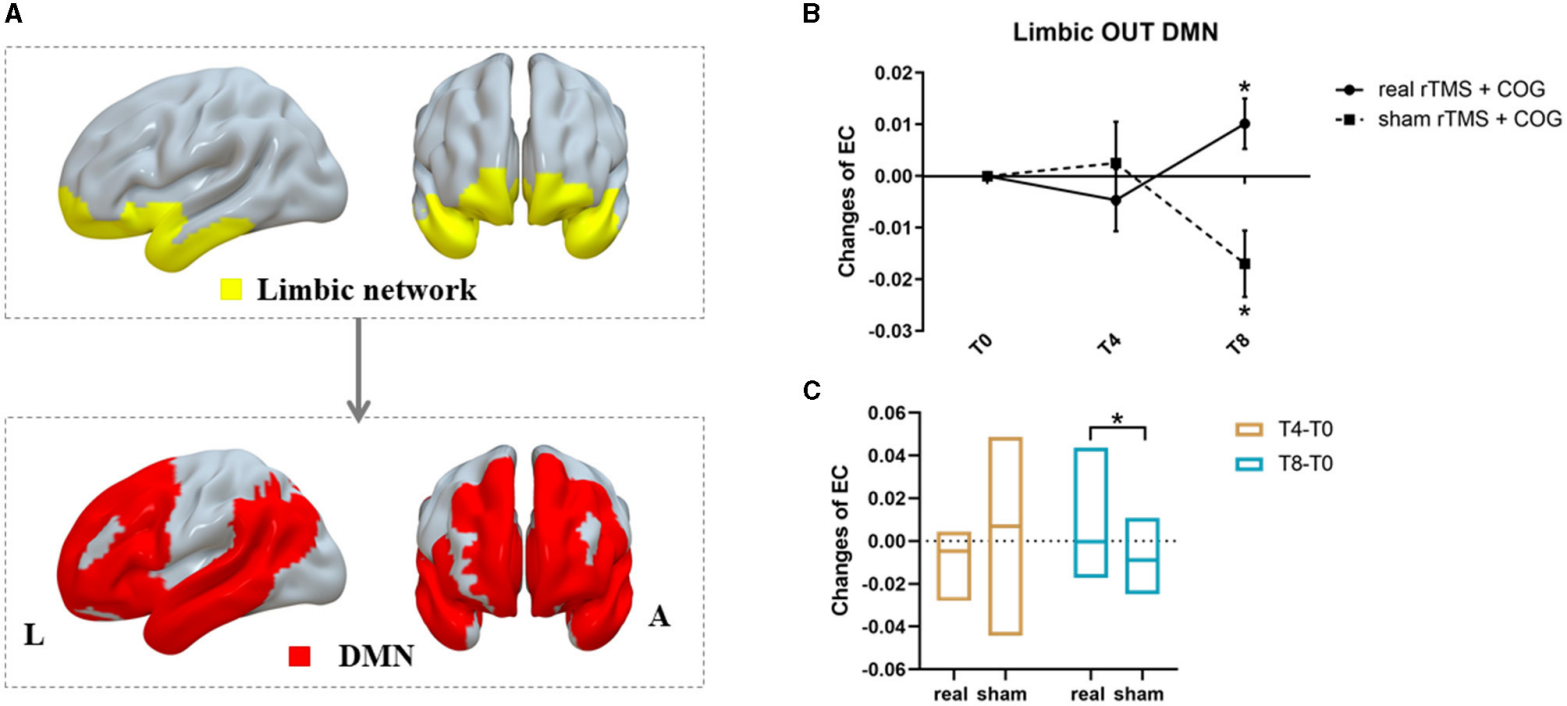
Results of inter-network EC changes among seven classic resting-state networks. **(A)** The pattern diagram showing significant pairwise EC changes from limbic network out to DMN. **(B, C)** Line and bar charts showing the *post-hoc* analysis results. *Indicates *p* < 0.05, FWE corrected. L, left; R, right; DMN, default mode network.

For pairwise EC changes of the seven networks, a significant time × group interaction was only observed from the limbic network out to the default mode network (DMN) (F = 7.070, *p* = 0.005, Figure 4A). Compared with the baseline, the high-frequency rTMS + COG induced no significant EC changes at T4 (*p* = 0.231), but it produced a significant increase at T8 (*p* = 0.034). The sham group showed no significant EC changes at T4 (*p* = 0.381), but it showed a significant decrease at T8 (*p* = 0.014) (Figure 4B). The EC changes between the real and sham groups showed significant differences at T8 (*p* = 0.005) but not at T4 (*p* = 0.255) (Figure 4C).

### Partial correlation analysis

For voxel-level EC changes, partial correlation analysis showed that the EC changes from the left DLPFC out to the left IPL, left SFG, left PoCG, left thalamus, and right parahippocampus were all positively correlated with ADL changes at T4 or T8 (*P* < 0.05), indicating that the reduction in EC induced by the real rTMS intervention improved the ability to perform activities of daily living. In particular, the decrease in EC from the left DLPFC out to the right parahippocampus was associated with a decrease in ADAS-cog score 4 weeks after the end of the treatment (T8), indicating that the reduction in EC from the left DLPFC out to the right parahippocampus was associated with improved cognitive function in patients with mild to moderate AD in the long term. For the network-level EC changes, the increase in EC from the limbic network out to the DMN at T8 was correlated with an increase in MoCA score after rTMS-COG treatment. Table 3 shows the partial correlation analysis results.

**Table 3:** Partial correlation analysis of effective connectivity changes and neuropsychological score changes.

**EC changes**	**Neuropsychological score changes**		**PCC**	***p***
DLPFC → L-IPL	ADL	T4-T0	0.725	0.027
	ADL	T8-T0	0.916	0.004
DLPFC → L-SFG	ADL	T4-T0	0.687	0.041
	ADL	T8-T0	0.939	0.002
DLPFC → L-PoCG	ADL	T8-T0	0.940	0.002
DLPFC → L-Thalamus	ADL	T4-T0	0.858	0.003
	ADL	T8-T0	0.971	0.000
DLPFC → R-parahippocampus	ADAS-cog	T8-T0	0.822	0.023
	ADL	T8-T0	0.762	0.047
LTL → L-OFC	AVLT	T4-T0	−0.712	0.032
	ADL	T8-T0	0.817	0.025
LTL → R-STG	ADL	T8-T0	0.871	0.011
Limbic network → DMN	MOCA	T8-T0	0.770	0.043

EC, effective connectivity; PCC, partial correlation coefficient; L, left; R, right; DLPFC, dorsal lateral prefrontal cortex; IPL, inferior parietal lobe; SFG, superior frontal gyrus; PoCG, postcentral gyrus; LTL, lateral temporal lobe; OFC, orbital frontal cortex; STG, superior temporal gyrus; ADL, activities of daily living; ADAS-cog, Alzheimer's Disease Assessment Scale-Cognitive Subscale; AVLT, auditory verbal learning test–HuaShan version; MoCA, Montreal Cognitive Assessment.

## Discussion

Using the left DLPFC and the left LTL as the stimulation targets, we explored the EC alterations after multisite rTMS-COG therapy and obtained three main results: first, at the voxel level, EC changes from the left DLPFC out to the left IPL and left SFG, as well as from the left LTL out to the left OFC had a significant group × time interaction effect. Second, at the network level, a significant interaction was identified on the EC increment from the limbic network out to the DMN. Third, the decrease in EC at the voxel level and the increase in EC at the network level were associated with improved ability to perform activities of daily living and cognition.

### rTMS-COG therapy-induced effective connectivity decrease from stimulation targets out to several brain regions at the voxel level

In our study, rTMS-COG therapy induced a decrease in EC from the left DLPFC out to the left IPL and from the left LTL out to the left OFC, while cognitive training alone (sham condition) induced a significant increase in EC in the long term (T8). rTMS stimulation on the left DLPFC has been observed to have definite benefits for patients with mild AD (Di Lazzaro et al., 2021). As an important node of the ECN and the FPN (Vincent et al., 2008), the left DLPFC has been mostly used and has been proven to be a beneficial stimulation target for AD (Cotelli et al., 2008, 2011; Ahmed et al., 2012). The IPL related to bottom-up attention and episodic memory is an important node of the ECN (Xiao et al., 2022). Information flow from the IPL to the anterior DMN subsystem has been reported, along with extensive connections between the parietal cortex and the frontal cortex (the parieto-frontal circuit) (Buckner et al., 2009; Luo et al., 2019). Our study found a significant decrease in EC from the left DLPFC to the ipsilateral IPL, associated with improved ADL score both in the short and long term (T4 and T8). This is also in line with previous observations that the TMS of the left DLPFC could selectively modulate functional connectivity both within and between the ECN and DMN in patients with depression (Liston et al., 2014). rTMS over DLPFC modulated the coupling between the ECN and the DMN in healthy people and in heroin-dependent individuals (van der Werf, 2010; Chen et al., 2013; Jin et al., 2022). Stimulation of the left LTL with transcranial direct current stimulation (tDCS) improved recognition memory in AD patients (Ferrucci et al., 2008). Similarly, in our study, a decrease in EC from the left LTL out to the ipsilateral OFC improved episodic memory in the short term (T4) and the ability to perform activities of daily living in the long term (T8).

For EC changes from the left DLPFC out to the left SFG, rTMS-COG therapy induced a significant decrease in EC in both the short and long terms, with no significant changes for cognitive training (sham condition). Causal interactions between the frontoparietal central executive and the DMN were well identified in a TMS/fMRI study (Chen et al., 2013). Since the FPN covers several frontoparietal areas, including the SFG (Vincent et al., 2008), our study showed that rTMS targeted on the left DLPFC also induces information flow within the FPN, resulting in benefits for ADL in both the short and long terms.

### rTMS-COG therapy-induced EC increase at the network level

rTMS is effective for increasing and decreasing (low-frequency: decrease, high-frequency: increase) functional coherence within the prefrontal-limbic network (Riedel et al., 2019). rTMS reportedly induces hypoconnectivity within the DMN in patients with amnestic mild cognitive impairment (aMCI)(Cui et al., 2019). However, the inter-network causal effect after multisite rTMS-COG treatment has yet to be expounded. In our study, compared with the cognitive training group (the sham condition), high-frequency rTMS-COG treatment induced a significant increase in EC from the limbic network out to the DMN at the 4-week follow-up after treatment (T8), indicating that multisite TMS-COG therapy had a long-term effect at the network level. Furthermore, the inter-network increase in EC was associated with cognition improvement (MoCA score increase).

It is particularly noteworthy that, although the cognitive training tasks were the same in the real rTMS and sham groups in our study, we could not ascribe the improvement in clinical outcomes in the real group to rTMS itself. It has been reported that TMS may have a synergistic effect with cognitive training (Rabey et al., 2013). Interlaced with cognitive training, rTMS has additional beneficial effects (Bentwich et al., 2011). Our results may aid the understanding of the neurobiological effects of rTMS-COG therapy on a macro-scale.

### Limitations

Our study has several limitations. First, due to the longitudinal design, the sample size and statistical efficiency were limited. Therefore, we cannot rule out the possibility that some of the changes we observed after rTMS-COG were not causally related to the treatment. Second, AD patients were recruited based on the NINCDS-ADRDA criteria instead of the amyloid/tau/neurodegenaration (ATN) diagnostic framework. These criteria were chosen because the cerebral spinal fluid (CSF) biomarker and molecular neuroimaging with positron emission tomography (PET) are usually unavailable in clinical practice due to their “invasive” characteristics. The heterogeneity across participants may have influenced the results, so our findings could be considered exploratory. When interpreting the findings of the present study, a great deal of caution is needed. A multi-center cohort study is needed to validate our pilot study.

## Conclusions

In summary, at the voxel level, multisite rTMS-COG therapy changed the EC from the stimulation targets out to several brain regions, covering important networks, especially the DMN, ECN, and FPC. At the network level, rTMS-COG increased the EC from the DMN out to the limbic network. All the alterations were accompanied by better outcomes related to the ability to perform activities of daily living and cognition function in the short or long terms or both. This study provides a novel explanation for the neurobiological mechanisms of multisite rTMS-COG therapy in AD patients and further sheds light on the direction of targeted brain network modulation in the future.

## Data availability statement

The raw data supporting the conclusions of this article will be made available by the authors, without undue reservation.

## Ethics statement

The study was approved by the Ethics Committee of Tongji Hospital (Wuhan, China). Written informed consent of all participants were obtained according to the declaration of Helsinki before enrollment. The patients/participants provided their written informed consent to participate in this study.

## Author contributions

YQ: conceptualization and writing. LB: data curation. FZ: visualization and investigation. SJ: software. TT: methodology, statistics, and software. MZ: supervision and validation. WZ: writing, reviewing, and editing. All authors contributed to the article and approved the submitted version.
